# Opto-CD28-REACT: optogenetic co-stimulatory receptor activation on non-engineered human T cells

**DOI:** 10.3389/fimmu.2025.1646135

**Published:** 2025-09-19

**Authors:** Anna K. Ehret, Sara Hartmann, Pavel Salavei, Virginia Andreani, Nicole Gensch, Laura Gámez-Díaz, Wolfgang W. Schamel

**Affiliations:** ^1^ Centre for Integrative Biological Signalling Studies (CIBSS), University of Freiburg, Freiburg, Germany; ^2^ Centre for Biological Signalling Studies (BIOSS), University of Freiburg, Freiburg, Germany; ^3^ Faculty of Biology, University of Freiburg, Freiburg, Germany; ^4^ Centre for Chronic Immunodeficiency (CCI), Medical Centre Freiburg and Faculty of Medicine, University of Freiburg, Freiburg, Germany; ^5^ Spemann Graduate School of Biology and Medicine (SGBM), University of Freiburg, Freiburg, Germany; ^6^ Core Facility Signalling Factory and Robotics, BIOSS, University of Freiburg, Freiburg, Germany; ^7^ Centre for Cell and Gene Therapy Freiburg (CGF), Medical Centre Freiburg and Faculty of Medicine, University of Freiburg, Freiburg, Germany

**Keywords:** non-engineered T cells, co-stimulatory receptor activation, T cell receptor, extracellular optogenetics, phytochromes

## Abstract

T-cell activation is a highly regulated process requiring both antigen recognition via the T-cell receptor (TCR) and co-stimulatory signaling, notably through the co-stimulatory receptor CD28. Here, we introduce an optogenetic platform for reversible and tunable full activation of human T cells that does not require genetic modification. We engineered opto-CD28-REACT, a recombinant protein comprising an anti-CD28 single-chain variable fragment, GFP, and phytochrome-interacting factor 6 (PIF6). This construct binds CD28 and thereby attaches PIF6 to CD28. Upon red light (630 nm) illumination, PIF6 binds to PhyB tetramer-coated beads, triggering CD28 signaling that can be attenuated by far-red light (780 nm) in 2 min. We show that opto-CD28-REACT synergizes with opto-CD3ϵ-REACT—a complementary optogenetic tool targeting the TCR complex—to induce light-dependent activation of both Jurkat cells and primary human T cells. Co-stimulation through both opto-REACT systems promotes ERK phosphorylation, upregulation of the activation markers CD69 and CD25, interleukin-2 (IL-2) secretion, and T-cell proliferation, reaching levels similar to conventional antibody-mediated stimulation. This strategy enables precise optical control over TCR and CD28 signaling in non-genetically modified T cells, offering a powerful approach for dissecting the regulatory dynamics of T-cell activation and advancing applications in synthetic immunology.

## 1 Introduction

T cells recognize antigens presented by antigen-presenting cells (APCs) through the T-cell receptor (TCR), leading to their activation, proliferation, and differentiation to execute immune functions.

The activation of T cells is governed by the TCR and co-receptor signaling pathways. The recognition of peptide antigens bound to major histocompatibility complex (MHC) molecules by the TCR alone is insufficient to induce a T-cell response in naive T cells, and instead results in non-responsiveness or T-cell anergy (1–3). For full activation, a second activation signal coming from co-stimulatory receptors is required. One example is the CD28 co-receptor binding to B7 ligands. CD28 is constitutively present on nearly all CD4^+^ T cells and a significant proportion of CD8^+^ T cells, at a density of 60,000 molecules per cell (4), compared to 20,000 TCR molecules (5). Co-receptor activation prevents anergy by modulating cell cycle progression and reducing cell death or apoptosis (6, 7). In addition, it also lowers the threshold for TCR activation by enabling an effective activation by few antigenic ligands (8–10). Numerous studies have shown that CD28 co-stimulation is important for initiating the signaling pathways of the activator protein 1 (AP-1), nuclear factor of activated T cells (NFAT), and nuclear factor kappa-light-chain-enhancer of activated B cells (NFκB). For example, CD28-mediated NFκB activation depends on protein kinase C θ and requires the interaction of growth factor receptor bound protein 2 (Grb2) with CD28 (11). Furthermore, Grb2 bound to CD28 cooperates with Vav1 (12, 13) to activate NFAT/AP-1-dependent transcription [e.g., interleukin-2 (IL-2)] (14). Even in the absence of TCR engagement, CD28 ligation can trigger NFAT and NFκB activation (15–18).

*In vivo* naive T cells are activated while they migrate. Pioneering studies using two-photon microscopy have revealed the dynamics of this process. In lymph nodes, T cells first form brief serial interactions with antigen-loaded APCs, followed by stable contacts. They then detach, increase their motility, and begin to proliferate (19–21). During the contact with the APC, the TCR and CD28 are stimulated, initiating signaling. Upon detachment, signaling quickly reverts, most likely because the interactions of the TCR and CD28 with their ligands are lost (22). To date, it is not well understood how the temporal patterns of ligand encounter shape T-cell activation and the differentiation process, since technologies to determine ligand-binding kinetics *in vitro* are scarce.

Optogenetics, a technology that enables precise control of molecular processes using light, is used to study the impact of ligand-binding kinetics on T-cell activation behavior *in vitro* (23–29). This non-invasive, precise method uses light-responsive proteins, such as plant photoreceptors, to manipulate ligand–receptor interactions with high temporal and spatial resolution. Specifically, these proteins change their conformation upon illumination with specific wavelengths of light, enabling reversible control of protein–protein interactions. In optogenetic systems, the fusion of photoreceptors such as phytochrome B (PhyB) and its PhyB-interacting factor (PIF) to cellular ligands and receptors, respectively, allows the spatiotemporal control of receptor activation.

For TCR studies, PhyB-PIF systems have been engineered to control TCR clustering and activation in a red-light-dependent manner (25, 27–29). In one approach, Yousefi and colleagues used a system in which PIF is fused to the ectodomain of the TCRβ chain and expressed by the T cell. Tetramers of the light-responsive protein PhyB act as the ligand. The PIF-TCR and PhyB interact upon red light (e.g., 630 nm) illumination and dissociate under far-red light (e.g., 780 nm) (29). This approach provides unprecedented resolution in regulating TCR signaling, allowing researchers to investigate the effects of the interaction dynamics on TCR signaling and T-cell activation. Furthermore, advancements in extracellular optogenetic techniques have extended these capabilities to non-genetically modified cells (30). A second approach utilizes this extracellular optogenetics. Here, the optogenetic tool (PIF) is not expressed by the T cell; instead, it is coupled to a single-chain variable fragment (scFv), which binds CD3; these reagents are called Light-inducible T-cell engager (LiTE) (23) and optogenetic Receptor Activation (opto-REACT) (24). Once LiTE or opto-REACT is bound to the TCR, PIF and PhyB interact when illuminated with red light, leading to TCR clustering and subsequent signaling activation. This interaction is terminated within seconds with far-red light illumination. For the sake of differentiation, we will henceforth refer to opto-REACT as opto-CD3ϵ-REACT.

In this study, we engineered the opto-CD28-REACT tool to fully activate non-engineered primary human T cells using optogenetics. We show that opto-CD28-REACT can be combined with opto-CD3ϵ-REACT to stimulate both receptors controlled by light. This system offers an extraordinary opportunity to control CD28- and CD3-mediated signaling with spatiotemporal precision, thereby dissecting the roles of TCR and CD28 in T-cell activation.

## 2 Results

### 2.1 Recombinant opto-CD28-REACT binds to CD28-positive cells

**Figure 1:**
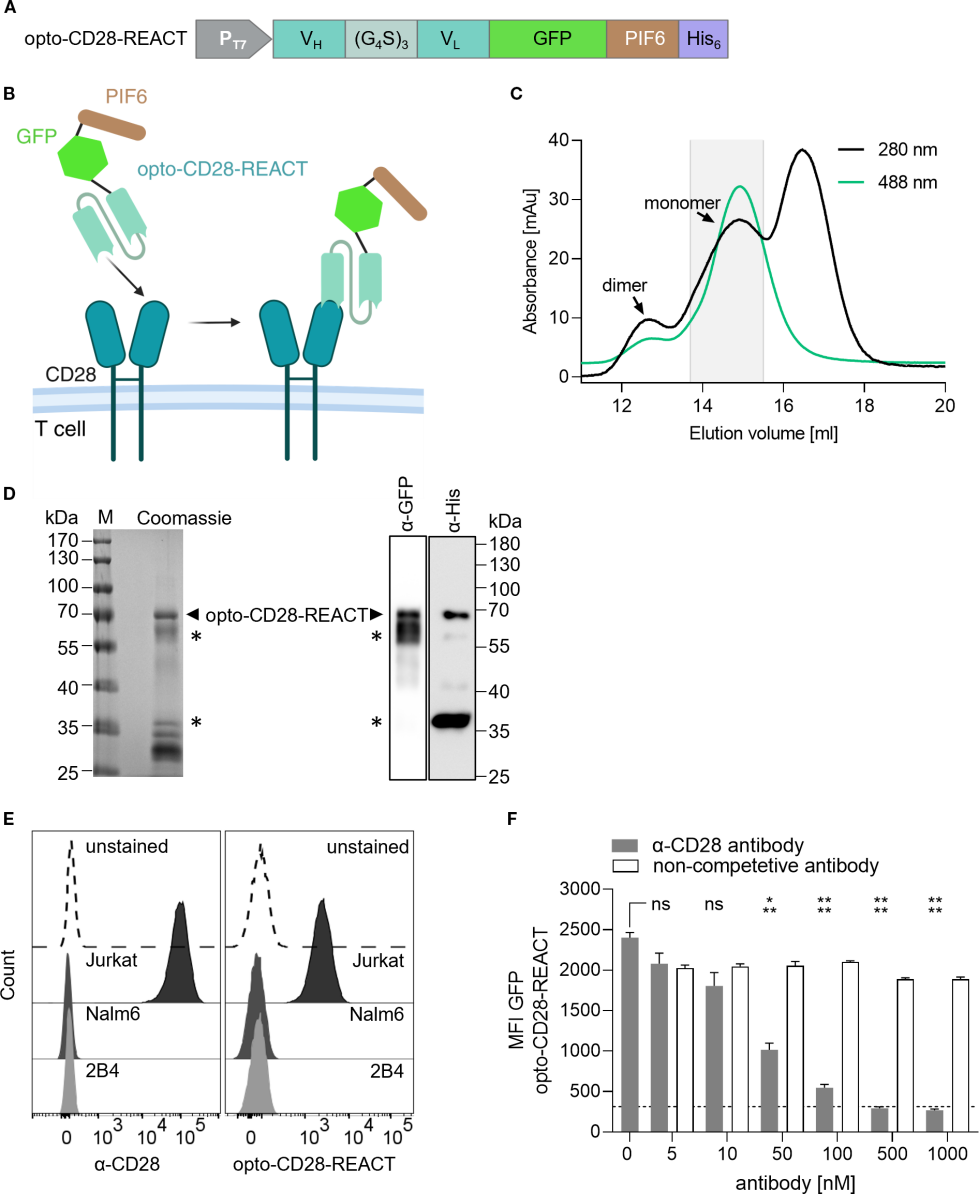
Production and CD28-binding of opto-CD28-REACT. **(A)** Design of opto-CD28-REACT. The fusion protein consists of a CD28.3-derived single-chain variable fragment (scFv), monomeric GFP (moxGFP), the first 100 amino acids of phytochrome-interacting factor 6 (PIF6), and a His6-tag. **(B)** Scheme of opto-CD28-REACT binding to the CD28 co-stimulatory receptor on T cells. **(C)** Purification of recombinantly expressed opto-CD28-REACT by size exclusion chromatography. Absorbance was monitored at 280 nm (total protein) and 488 nm (GFP). Peaks of monomeric and dimeric proteins are marked. The gray box indicates collected and pooled fractions. **(D)** On the left side, SDS-PAGE and Coomassie staining of pooled fractions are shown. On the right side, a Western blot developed with anti-GFP and anti-His antibodies is depicted. Purified opto-CD28-REACT is seen with a molecular weight of 67 kDa. **(E)** Human T cells (Jurkat), human B cells (Nalm6), and murine T cells (2B4) were incubated with an Alexa Fluor 488 (AF488)-coupled anti-human CD28 antibody (1:200, left panel) and opto-CD28-REACT (100 nM, right panel). As control, Jurkat cells were left unstained. After washing, AF488 (anti-CD28) and GFP (opto-CD28-REACT) fluorescence intensities were recorded by flow cytometry. *n* = 3, each in technical triplicates. **(F)** Jurkat T cells were incubated simultaneously with 100 nM opto-CD28-REACT and increasing concentrations of an anti-CD28 or a non-competitive antibody. As control, Jurkat cells were left unstained (dashed line). After washing, the GFP (opto-CD28-REACT) fluorescence intensity was recorded by flow cytometry. *n* = 3, each in technical triplicates. Samples treated with increasing concentrations of anti-CD28 antibody were compared to cells, which were only treated with opto-CD28-REACT for statistical analysis. Error bars represent SEM. MFI, median fluorescence intensity. ns: p > 0.05; *: p < 0.05; **: p < 0.01.

We developed a system for the reversible co-stimulation of non-genetically modified human T cells utilizing light-controlled co-receptor CD28 clustering. This system compromises a recombinant engineered protein called opto-CD28-REACT. It contains an scFv based on the anti-human CD28 antibody CD28.3, followed by the green fluorescent protein (GFP) variant moxGFP (31) (GFP hereinafter), the first 100 amino acids of phytochrome-interacting factor 6 (PIF6) carrying mutations in C9S and C10S (29), and a His6-tag for purification (**Figures 1A, B**, **Supplementary Figure S1A**). This design is similar to a previous successful one targeting the TCR (24), here called opto-CD3ϵ-REACT. After expression in *Escherichia coli*, the engineered protein was purified using Ni^2+^ affinity chromatography via its His6-tag, and the monomeric form of opto-CD28-REACT was subsequently obtained by size-exclusion chromatography (**Figure 1C**, collected fractions are highlighted in gray). Successful purification of opto-CD28-REACT was confirmed via sodium dodecyl sulfate–polyacrylamide gel electrophoresis (SDS-PAGE), Coomassie staining, and Western blotting with anti-His and anti-GFP antibodies, showing a band at the expected molecular weight of 67 kDa (**Figure 1D**, arrowheads). We also obtained bands of lower molecular weight likely corresponding to scFv-GFP and PIF6-His proteins (**Figure 1D**, asterisks). Flow cytometry data (**Figure 1E**) demonstrate that opto-CD28-REACT binds to the human CD28-expressing Jurkat cell line. In contrast, no binding to the human CD28-negative Nalm6 B cell line or the murine 2B4 T cell line was observed, confirming the binding specificity of opto-CD28-REACT. Furthermore, specific binding to human CD28 was confirmed by a competition experiment in which the binding of opto-CD28-REACT to Jurkat cells could be prevented by an established anti-CD28 antibody. With increasing concentration of the anti-CD28 antibody, a decrease in GFP median fluorescence intensity (MFI) of the opto-CD28-REACT is seen (**Figure 1F**). Finally, a dose-response binding analysis (**Supplementary Figure S1B**) further characterizes binding of opto-CD28-REACT to CD28-expressing Jurkat cells.

### 2.2 Opto-CD28-REACT stimulates the co-receptor CD28 in a reversible light-dependent manner

**Figure 2:**
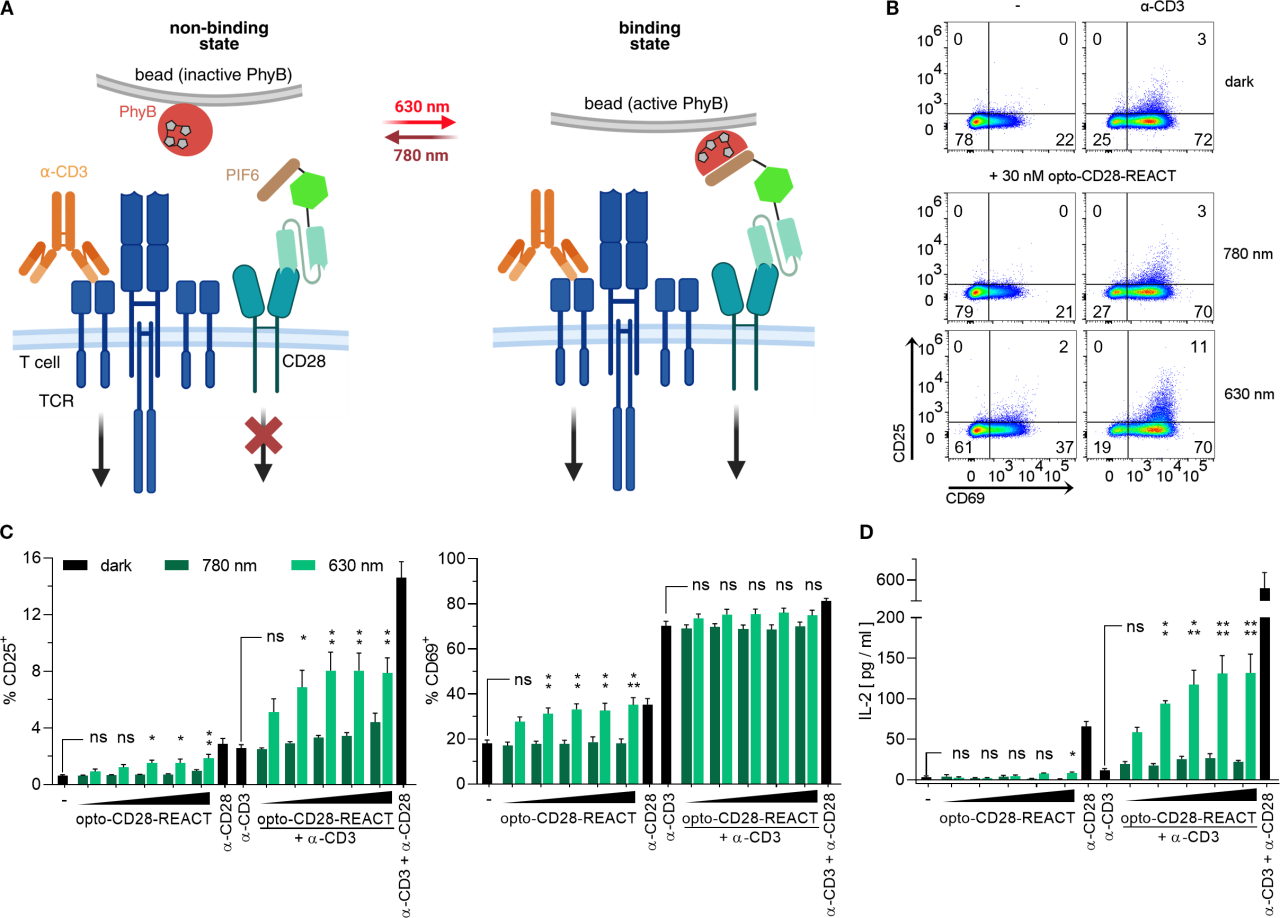
Optogenetic activation of the co-stimulatory receptor CD28 in Jurkat T cells. **(A)** Scheme of CD28 activation using opto-CD28-REACT. Clustering of CD28 is induced by binding of PIF6 to oligomerized PhyB on beads in response to 630-nm light and the binding is terminated by 780-nm illumination. TCR clustering is induced by anti-CD3 antibody binding. **(B)** Jurkat cells treated with 30 nM opto-CD28-REACT and with or without anti-CD3 antibody were co-cultured with PhyB coupled beads. Cells were illuminated for 24 h (for the illumination pattern, see the Methods section). As controls, Jurkat cells were left unstimulated (−) or stimulated with anti-CD3 antibodies in the dark. Subsequently, cells were stained with anti-CD25-PE and anti-CD69-AF647 antibodies and analyzed by flow cytometry. Dot plots show CD69 and CD25 expression. **(C)** Bar diagrams of the experiment from **(B)** displaying percent of CD25^+^ and CD69^+^ cells. Concentration of opto-CD28-REACT increasing from left to right: 0, 5, 10, 30, 50, and 100 nM. As controls, Jurkat cells were left unstimulated (−) or stimulated with anti-CD28 or anti-CD3 antibodies separately or in combination in the dark. **(D)** Supernatants of **(C)** were analyzed for IL-2 secretion by ELISA. All experiments depicted in this figure are *n* = 2, each in technical triplicates. Samples treated with increasing opto-CD28-REACT concentrations and 630-nm light were compared to unstimulated cells for statistical analysis. Samples treated with increasing opto-CD28-REACT concentrations, 630-nm light, and anti-CD3 antibodies were compared to cells treated with only anti-CD3 antibodies for statistical analysis. Error bars represent SEM. ns: p > 0.05; *: p < 0.05; **: p < 0.01.

Our optogenetic co-receptor activation is based on the light-dependent interaction of PhyB with PIF6. In our system, PIF6 of the CD28-bound opto-CD28-REACT interacts with PhyB coupled to streptavidin-coated beads upon illumination with red (630 nm) light (**Figure 2A**). This light-induced interaction leads to CD28 clustering and subsequent activation of the downstream signaling pathways. In contrast, illumination with far-red (780 nm) light leads to dissociation of the PhyB–PIF6 interaction, thereby terminating CD28 clustering.

To test the function of opto-CD28-REACT, Jurkat T cells were loaded with this reagent and stimulated for 24 h with PhyB-coupled beads, determining the PhyB–PIF6 interaction by light (**Figure 2B**). As controls, the cells were left unstimulated without opto-CD28-REACT and PhyB (- dark) or stimulated with anti-CD3 antibodies (α-CD3 dark). Cell activation was assessed by flow cytometry analysis of CD25 and CD69 expression (**Supplementary Figure S2**). Optogenetic activation of CD28 slightly upregulated CD25 and CD69 with the 630-nm light, but not with the 780-nm light when compared to the untreated cells (**Figure 2C**). Cells treated with increasing concentrations of opto-CD28-REACT ranging from 5 to 100 nM responded with a concentration-dependent increase of CD25 and CD69 expression when illuminated only with the 630-nm light (**Figure 2C**). This demonstrates that the light-dependent stimulation of the co-receptor CD28 can be achieved with our opto-CD28-REACT reagent.

To test for cooperation between CD28 and TCR in our system, we also stimulated cells with anti-CD3 antibodies together with opto-CD28-REACT (**Figure 2B**). Stimulation with anti-CD3 alone resulted in the upregulation of CD25 and CD69 expression. Importantly, co-stimulation with opto-CD28-REACT together with anti-CD3 resulted in stronger CD25 and CD69 upregulation with the 630-nm but not with the 780-nm light, compared to anti-CD3 stimulation alone (**Figure 2C**).

Optogenetic stimulation of CD28 had an even more pronounced effect when IL-2 production of the T cells was assessed. Only when cells were treated with both anti-CD3 and opto-CD28-REACT under illumination of the 630-nm light was a significant concentration-dependent increase in IL-2 secretion observed (**Figure 2D**). Notably, the amount of IL-2 produced by stimulating via both receptors was higher than the sum of the single stimulated cells (32, 33).

Altogether, these data confirm that opto-CD28-REACT is functional and can synergize with TCR signals to enhance T-cell activation.

**Figure 3:**
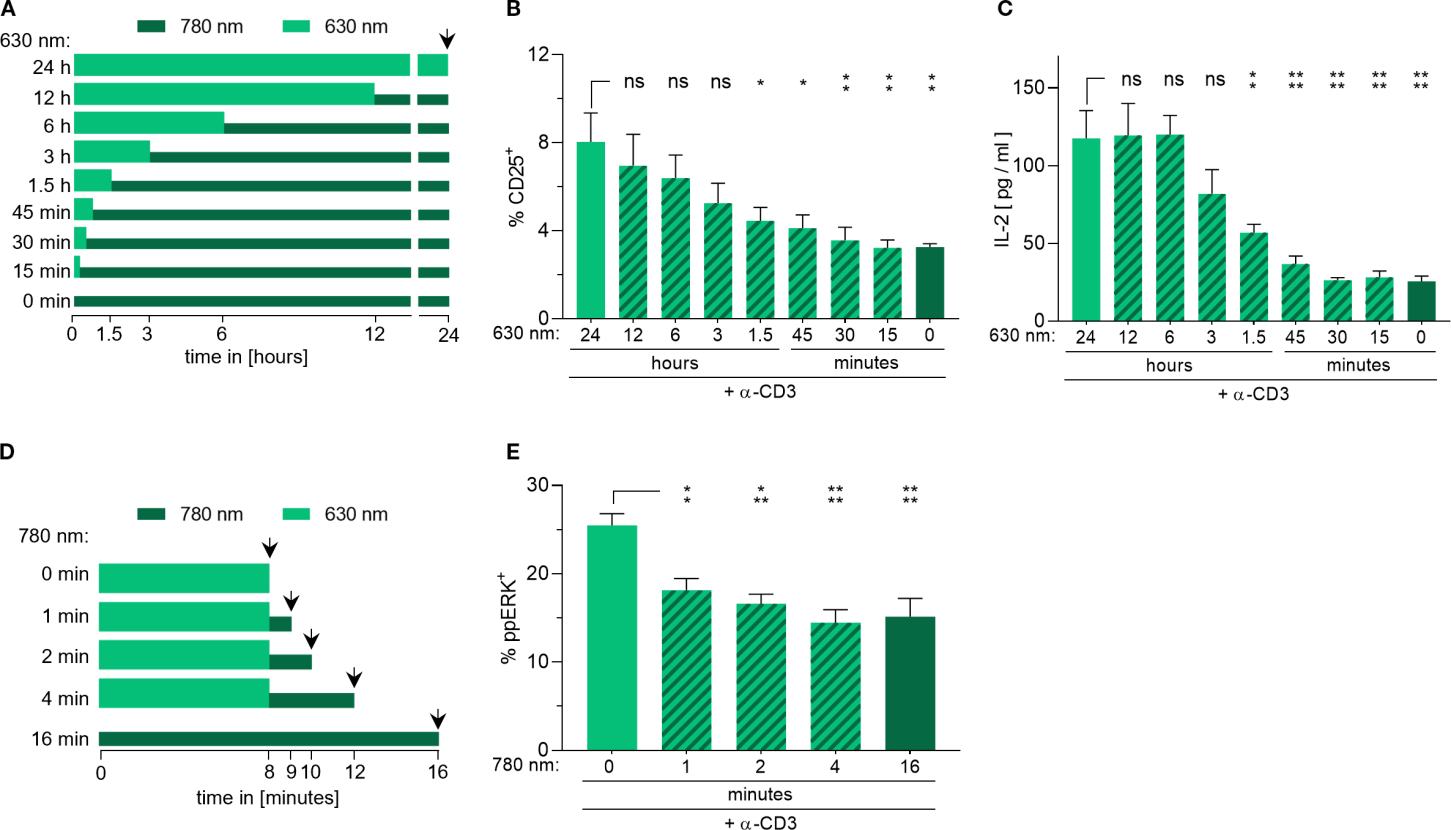
Reversible modulation of CD28 co-stimulatory signaling using the opto-CD28-REACT. **(A)** Schematic representation of the experiments shown in **(B, C)**. The total experimental time was 24 h when measurement occurred (arrowhead). The thick, light green bars indicate the time cells were illuminated with pulsatile 630 nm light. This was followed by a period of pulsed 780-nm light, which is indicated by the thin, dark green bars. **(B)** Jurkat cells were stimulated with 30 nM of opto-CD28-REACT using the light regimes of **(A)**. Cells were stained with an anti-CD25-PE antibody and analyzed by flow cytometry. **(C)** Supernatants of **(B)** were analyzed for IL-2 secretion via ELISA. *n* = 2, each in technical triplicates. **(D)** Schematic representation of the experiment shown in **(E)**. The thick, light green bars indicate the time cells were illuminated with the pulsatile 630-nm light in the beginning. This was followed by a period of pulsed 780-nm light, which is indicated by the thin, dark green bars. Measurement was performed at the arrowheads. **(E)** Jurkat cells were stimulated with 30 nM of opto-CD28-REACT using the light regimes of **(D)**. Cells were stained with an anti-phospho-ERK antibody and analyzed by flow cytometry. *n* = 4, each in technical triplicates. Samples treated with the 780-nm light were compared to samples only treated with the 630-nm light for statistical analysis. Error bars represent SEM. ns: p > 0.05; *: p < 0.05; **: p < 0.01.

To test the reversibility of opto-CD28-REACT stimulation, Jurkat cells loaded with opto-CD28-REACT and co-cultured with PhyB-coated beads were illuminated with the 630-nm light for a given amount of time followed by illumination with 780 nm for the remainder of a 24-h period (**Figure 3A**). Throughout the experiment, cells of all conditions were simultaneously stimulated via the TCR using a low concentration of an anti-CD3 antibody. Both CD25 expression (**Figure 3B**) and IL-2 secretion (**Figure 3C**) show a gradual decrease with an increase in the duration of the 780-nm light exposure. This suggests that the opto-CD28-REACT enables reversible control of CD28 activation.

Next, we wanted to examine how rapidly CD28 signals are turned off. To this end, we assessed the dynamics of extracellular signal-regulated kinase (ERK) phosphorylation after ligand detachment from CD28. Opto-CD28-REACT-loaded Jurkat T cells were co-cultured with opto-APCs, which are adherent human embryonic kidney (HEK) 293 cells loaded with PhyB (27). Stimulation of the TCR was carried out by the addition of a low concentration of an anti-CD3 antibody. Cells were first treated for 8 min with the 630-nm light and then the interaction between PhyB and opto-CD28-REACT was terminated by 780-nm light illumination (**Figure 3D**). After certain periods of time, cells were stained with an anti-phospho-ERK antibody and measured by flow cytometry. One minute after the termination of ligand binding to CD28, a significant decrease in the phospho-ERK positive cells can already be seen (**Figure 3E**, **Supplementary Figure S3**). After 2 min, the percentage of cells was at the level of cells that were not stimulated via CD28 (dark green bar), indicating that the CD28 signal was gone. We conclude that after ligand detachment, CD28 signals can be turned off in 2 min.

### 2.3 Optogenetic co-stimulation of the TCR and CD28 enhances T-cell responses

**Figure 4:**
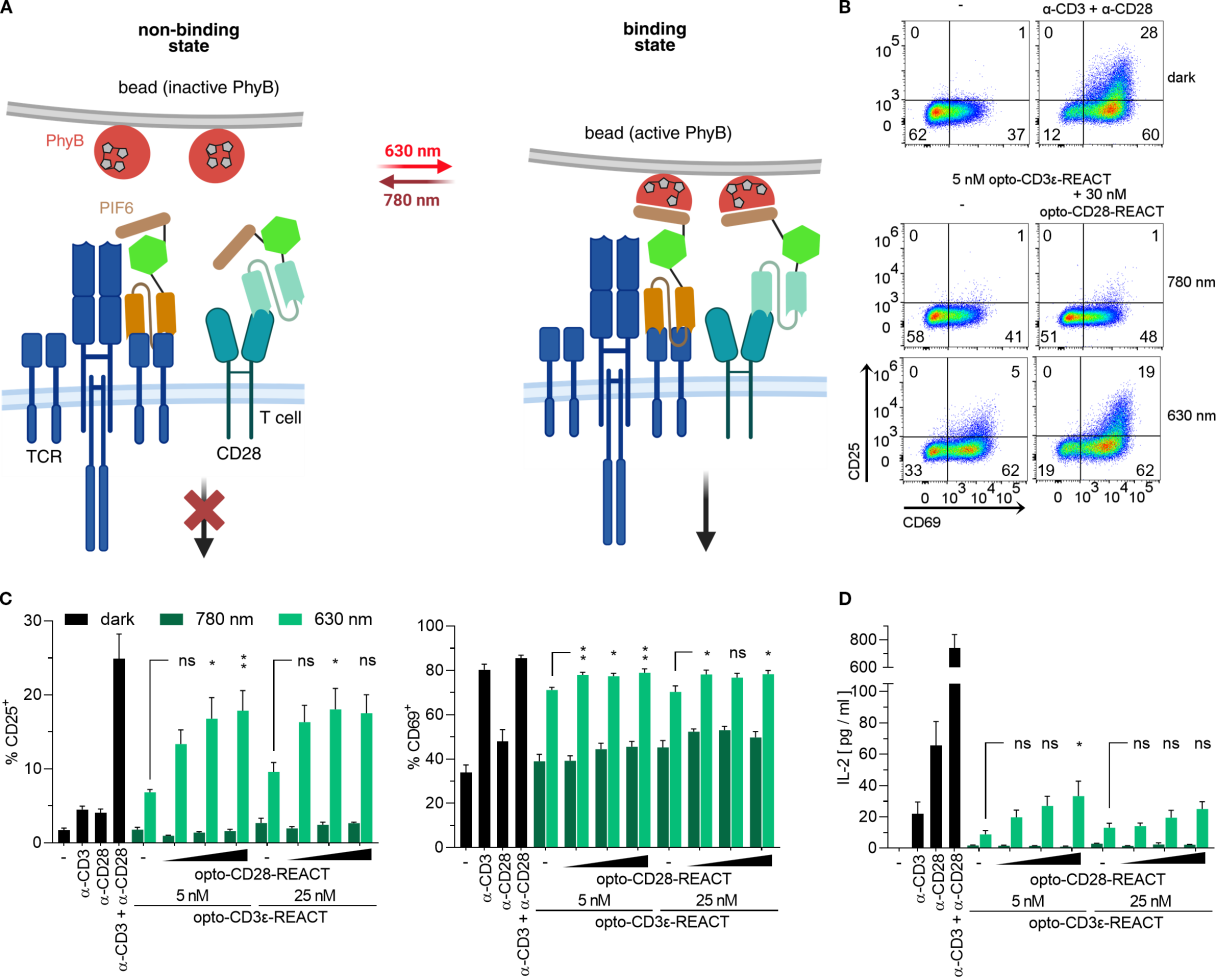
Light-based activation of TCR and CD28 in Jurkat T cells. **(A)** Scheme of simultaneous TCR and CD28 activation under optogenetic control. Under 780-nm light, PhyB binds neither to opto-CD3ϵ-REACT nor to opto-CD28-REACT, thereby preventing TCR and CD28 signaling. With 630-nm light illumination, PhyB binds to PIF6, leading to clustering of TCR and CD28 and subsequent signaling. **(B)** Jurkat cells treated with 5 nM opto-CD3ϵ-REACT and with or without 30 nM opto-CD28-REACT were co-cultured with PhyB-loaded beads. Cells were illuminated for 24 h (for the illumination pattern, see the Methods section). As controls, Jurkat T cells were left untreated (−) or stimulated with anti-CD28 and anti-CD3 antibodies in the dark. Cells were stained with anti-CD25-PE and anti-CD69-AF647 antibodies and measured by flow cytometry. Dot plots show CD69 and CD25 expression. **(C)** Bar diagrams show the percentage of CD25^+^ (left panel) and CD69^+^ (right panel) of the experiment from **(B)**. Concentration of opto-CD28-REACT increases from left to right: 0, 10, 30, and 50 nM. Two concentrations of opto-CD3ϵ-REACT were used (5 and 25 nM). As controls, Jurkat T cells were left untreated (−), stimulated with anti-CD28 or anti-CD3 antibodies separately or in combination in the dark. **(D)** Supernatants of **(C)** were analyzed for IL-2 secretion via ELISA. All experiments depicted in this figure are *n* = 2, each in technical triplicates. Samples treated with increasing opto-CD28-REACT concentrations and 630-nm light were compared to respective opto-CD3ϵ-REACT concentrations-only conditions for statistical analysis. Error bars represent SEM. ns: p > 0.05; *: p < 0.05; **: p < 0.01.

To test the combined effect of the two opto-REACTs in regulating T-cell activation, we used both opto-CD28-REACT and opto-CD3ϵ-REACT (24) to enable simultaneous, light-dependent clustering of CD28 and the TCR using PhyB-coupled beads (**Figure 4A**). Jurkat T cells were stimulated with both opto-REACTs and cultured with PhyB-coupled beads for 24 h. Under far-red illumination (780 nm), the T cells did not upregulate CD69 or CD25 compared to unstimulated cells (- dark) (**Figure 4B**). However, upon exposure to red light (630 nm), cells significantly upregulated CD25 and CD69 expression, demonstrating successful optogenetic co-stimulation via CD28 and TCR (**Figure 4B**).

To determine whether opto-CD28-REACT and opto-CD3ϵ-REACT were both active when administered in combination, a titration of both was performed. To this end, Jurkat T cells were stimulated with two concentrations of opto-CD3ϵ-REACT (5 and 25 mM) and three concentrations of opto-CD28-REACT (10, 30, and 50 nM). An opto-CD28-REACT concentration-dependent increase in CD25 and CD69 expression (**Figure 4C**) as well as in IL-2 secretion (**Figure 4D**) was observed under the 630-nm light but not when cells were illuminated with the 780-nm light. Higher concentrations of opto-CD3ϵ-REACT (25 nM) show no increase of any of the readouts compared to the lower concentration (5 nM) (**Figures 4C, D**). Furthermore, no difference in stimulation between 30 and 50 nM of opto-CD28-REACT was observed. Based on these data, 5 nM of opto-CD3ϵ-REACT and 30 nM of opto-CD28-REACT were used for further experiments. These results highlight the combined use of both opto-REACTs as a robust and tunable platform for light-controlled TCR and CD28 receptor activation.

### 2.4 Optogenetic stimulation of the TCR and CD28 leads to light-dependent activation of primary human T cells

**Figure 5:**
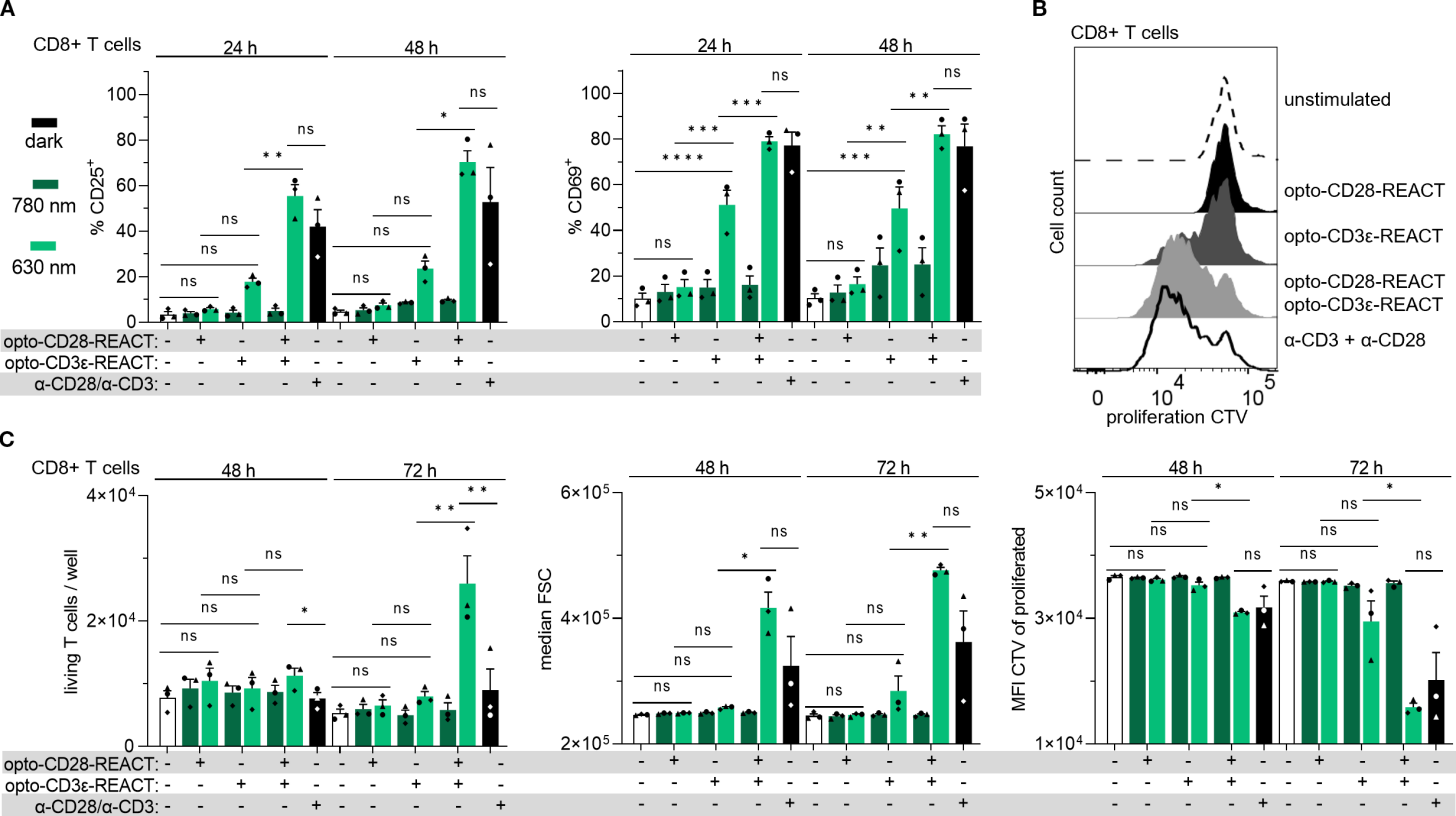
Primary human CD8^+^ T cells are activated and proliferate upon stimulation with opto-CD28- and opto-CD3ϵ-REACT. Primary T cells from human blood were treated with 5 nM opto-CD3ϵ-REACT and 30 nM opto-CD28-REACT separately or in combination. Cells were illuminated for 24, 48, and 72 h (the illumination protocol is provided in the Methods section). As controls, cells were either left untreated (−) or treated with anti-CD3 and anti-CD28 antibodies. Controls were not illuminated with light. After 24, 48, and 72 h of stimulation, cells were stained with zombie NIR, anti-CD3-AF488, anti-CD8-AF700, anti-CD25-PE, and anti-CD69-AF647 antibodies for subsequent flow cytometry analysis. The gating strategy is shown in **Supplementary Figure S3**. **(A)** Bar diagrams show the percent of CD25^+^ (left panel) and CD69^+^ (right panel) of the CD8^+^ living T cells after 24 and 48h. **(B)** The flow cytometry histograms show the proliferation of the living CD8^+^ T cells stained with CellTrace Violet (CTV). Cells were stimulated as given using pulsed 630-nm light and in the dark for the controls for 72h. **(C)** The bar diagrams show the numbers of living CD8^+^ T cells per well, the size of the CD8^+^ T cells (median FSC), and the MFI of the proliferated CD8^+^ cells after 48 and 72h All experiments depicted in this figure are *n* = 3 healthy donors, each in technical duplicates. All 630-nm-treated, unstimulated, and antibody-treated samples of every time point were compared with every other one for statistical analysis. Error bars represent SEM. FCS, forward scatter; CTV, CellTrace Violet; MFI, median fluorescence intensity. ns: p > 0.05; *: p < 0.05; **: p < 0.01; ****: p < 0.0001.

Having shown that Jurkat cells can be optogenetically activated using opto-CD3ϵ-REACT and opto-CD28-REACT, we next tested the ability of these reagents to activate primary human T cells. To this end, peripheral blood human T cells were isolated from healthy donors using Ficoll density gradient centrifugation. Then, the cells were incubated with the opto-REACT reagents, allowing binding to their specific receptor, without the need for any genetic modification. Optogenetic activation was achieved by co-incubating the cells with PhyB-coupled beads over a period of 3 days, during which the cells were treated with either the 630-nm or the 780-nm light. After 24 and 48 h, CD25 and CD69 expression was upregulated in cells stimulated with both opto-REACTs under the 630-nm but not under the 780-nm light compared to the unstimulated control. This was observed in both CD8^+^ (**Figure 5A**, **Supplementary Figures S4**, **S5**) and in CD8^−^ (**Supplementary Figure S6A**) T cells. The extent of cell activation using the opto-REACTs was similar to the one induced by conventional stimulation with anti-CD3 and anti-CD28 antibodies. Optogenetic stimulation of CD28 alone led to minimal upregulation of CD25 or CD69 expression, while TCR alone led to moderate upregulation. However, co-stimulation via both opto-REACTs showed a synergistic response between CD28 and TCR signaling. This demonstrates that both optogenetic stimuli can be combined generating an effective activation, although they both bind to the same PhyB ligand.

Strong synergy between opto-CD28-REACT and opto-CD3ϵ-REACT is also observed in their capacity to induce proliferation of CD8^+^ T cells after 48 and 72 h of stimulation, as demonstrated by the dilution of the proliferation dye CellTrace Violet (CTV) (**Figures 5B, C**). This proliferative response is in line with the increase in cell size upon stimulation as detected by the forward scatter and side scatter values and the percentage of living cells (**Figure 5C**, **Supplementary Figure S5B**). Similar trends were observed in CD8^−^ cells (**Supplementary Figures S6B, C**). Furthermore, the ratio of CD8^+^ and CD8^−^ was not influenced by the stimulation conditions (**Supplementary Figure S6D**).

**Figure 6:**
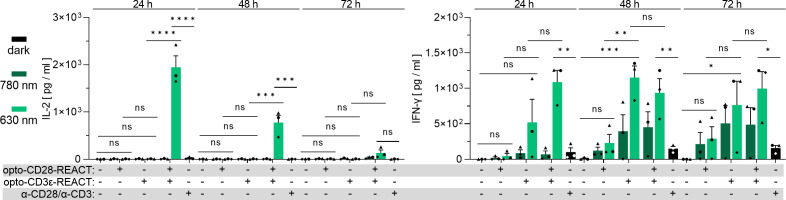
Primary human T cells secrete cytokines upon stimulation with opto-CD28- and opto-CD3ϵ-REACT. The cell culture supernatants of the stimulated and control primary human T cells from the experiments in **Figure 5** and **Supplementary Figure S5** were analyzed for IL-2 and IFN-γ secretion by ELISA. *n* = 3 healthy donors, each in technical duplicates. All 630-nm-treated, unstimulated, and antibody-treated samples of every time point were compared with every other one for statistical analysis. Error bars represent SEM. ns: p > 0.05; *: p < 0.05; **: p < 0.01; ****: p < 0.0001.

To evaluate functional activation, we quantified the amount of the secreted cytokines IL-2 and interferon-γ (IFN-γ) in the medium of the stimulated T cells (including the CD8^+^ and CD8^−^ cells) by enzyme-linked immunosorbent assay (ELISA). As expected, light-dependent secretion of both cytokines was observed (**Figure 6**). Only when cells were treated with both opto-REACTs under the 630-nm light was IL-2 detected after 24 h, followed by a decline over time. IFN-γ secretion was also observed upon combined stimulation via CD28 and TCR, and to a lesser extent by only opto-CD3ϵ-REACT, starting at 24 h and increasing over time. The low cytokine secretion in the antibody control could be explained by the experimental setup. For the optogenetic stimulations, PhyB was loaded onto beads, whereas the antibodies were coated to the plastic plates. In conclusion, these results confirm that primary human T cells can be optimally and synergistically activated using opto-CD3ϵ-REACT and opto-CD28-REACT in combination.

### 2.5 Supplementing additional opto-REACTs and PhyB-coupled beads during prolonged T-cell stimulation does not increase cell activation but reduces viability

**Figure 7:**
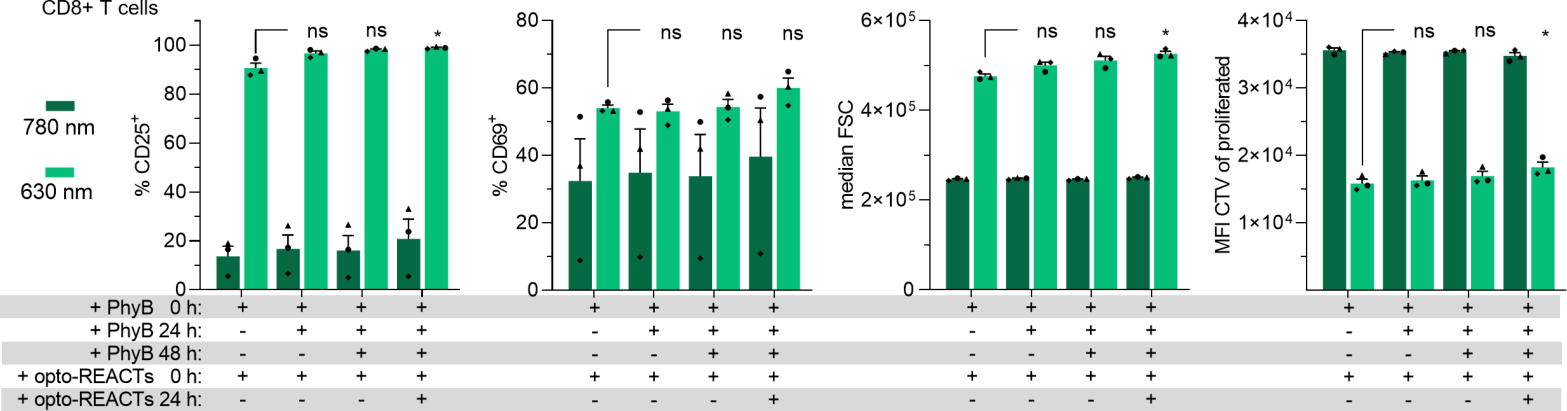
Adding of additional opto-REACTs and PhyB does not enhance activation of CD8^+^ primary T cells. PBMCs were treated with 30 nM opto-CD28-REACT and 5 nM opto-CD3ϵ-REACT at the start of the experiment and co-cultured with PhyB-loaded beads. Cells were illuminated for 72 h (the illumination protocol is provided in the Methods section). Fresh PhyB-loaded beads were added after 24 or 48 h to the wells that initially received beads at 0 h, as indicated. Additionally, after 24 h, both opto-REACTs (30 and 5 nM, respectively) were added again as indicated. After 72 h, cells were harvested and stained for flow cytometry as described in **Figure 5**. Bar diagrams of CD8^+^ T cells show, from left to right, the percentage of CD25^+^ cells and CD69^+^ cells, cell size (median FSC), and the MFI of proliferated cells. All experiments depicted in this figure are *n* = 3 healthy donors, each in technical duplicates. Samples treated with 630-nm light and where either PhyB or opto-REACTs were added after certain time periods were compared to conditions where PhyB and opto-REACTs were only added in the beginning of the experiment for statistical analysis. Error bars represent SEM. FCS, forward scatter; CTV, CellTrace Violet; MFI, median fluorescence intensity; SEM, standard error of the mean. ns: p > 0.05; *: p < 0.05.

Since PhyB activity is sensitive to oxidation and the opto-REACT reagents may be consumed by the T cells over time, this could impact prolonged stimulation periods, such as 72 h. To address this, we tested whether stimulation of primary human T cells could be enhanced by adding additional opto-REACTs and fresh PhyB-coupled beads after 24 or 48 h, when analysis of the cells was set at 72 h. The addition of PhyB after 24 and 48 h in CD8^+^ (**Figure 7**) and CD8^−^ (**Supplementary Figure S7**) cells resulted in a slight increase of the percentage of CD25^+^ cells and the cell size, whereas the percentage of CD69^+^ cells did not change (**Figure 7**). In the CD8^−^ cells, the percentage of CD69^+^ cells was slightly reduced when PhyB-coupled beads and opto-REACTs were additionally added (**Supplementary Figure S7A**). Both CD8^+^ and CD8^−^ cells proliferated weaker with the addition of the stimulants (**Figure 7**, **Supplementary Figure S7A**), and the total cell numbers also decreased (data not shown). This dataset indicates that the addition of opto-REACTs and PhyB-coupled beads may lead to overstimulation and increased cell death. Therefore, we do not recommend adding additional opto-REACTs or PhyB-coupled beads after 1 or 2 days of stimulation.

## 3 Discussion

The present study introduces opto-CD28-REACT as an innovative optogenetic tool for reversible and tunable co-stimulation via CD28 of non-genetically modified human T cells. This approach uses light-controlled CD28 clustering to induce CD28 signaling pathways essential for T-cell activation. While prior research has shown the advantage of using optogenetics to control TCR signaling (23–25, 27, 29), our study expands this principle to the co-receptor CD28.

We show that opto-CD28-REACT specifically binds to human CD28, thereby allowing CD28 to be stimulated by PhyB tetramers in a light-dependent manner. Stimulation was shown by downstream read-outs of CD28 signaling either by CD28 stimulation alone or in combination with the TCR, using anti-CD3 antibodies or opto-CD3ϵ-REACT. The simulation of CD28 alone appeared to be very low under opto-CD28-REACT and anti-CD28 antibody conditions. This is explained by the co-stimulatory properties by which CD28 enhances TCR signaling but has only weak outcomes on its own. The ability to trigger and terminate CD28 signaling in a light-dependent manner enables precise temporal (as shown here) and potentially spatial control with excellent resolution.

Controlling receptor activation without genetic modifications, such as using opto-CD28-REACT, has the advantage of enabling precise and reversible modulation of immune cell activity without the need for permanent alterations to the genome.

Our results reinforce the fundamental role of CD28 in modulating TCR signaling (34–36). CD28 co-stimulation enhances TCR-mediated responses, increasing IL-2 production and proliferation. Indeed, we observed that stimulation with opto-CD28-REACT in combination with anti-CD3 or opto-CD3ϵ-REACT stimulation led to stronger upregulation of CD69 and CD25 compared to the TCR stimulation alone. A similar synergistic effect was observed when evaluating secretion of IL-2 and IFN-γ, highlighting the necessity of co-stimulatory signals for optimal T-cell activation. Furthermore, it shows that the competition of both opto-REACTs for the binding to the PhyB molecules on the beads does not prevent simultaneous stimulation of both receptors. Our data suggest a saturation threshold beyond which increasing opto-CD3ϵ-REACT or opto-2CD28-2REACT concentrations did not further enhance activation. This could be due to a saturation of receptor binding or could align with previous studies indicating that optimal T-cell activation relies on balanced receptor engagement rather than excessive stimulation (37–39).

The reversibility of our optogenetic tools by alternating the wavelength between 630 and 780 nm enables precise temporal modulation of T-cell activation, distinguishing our optogenetic system from traditional antibody-based approaches that lack such reversibility. This advancement will enable us to study how the dynamics of receptor stimulation and, in this case, CD28 stimulation affects cell responses. In fact, studying the dynamics of T-cell activation is a new emerging research topic (28, 29, 40–42).

In addition, we show that the effect of CD28 co-stimulation on TCR-mediated activation events, such as upregulation of activation markers, cytokine secretion, or proliferation, was very similar in both CD8^+^ T cells, which are the cytotoxic T cells, and CD8^−^ T cells, which are mostly the CD4^+^ helper T cells. Further studies could explore the effects of CD28 stimulation using opto-CD28-REACT in different T-cell subsets, including regulatory T cells or γδ T cells, which have distinct co-stimulatory requirements (43–45).

In summary, this study presents a novel, reversible, and tunable optogenetic system for CD28-mediated T-cell co-stimulation, demonstrating its functional synergy with TCR signaling. The successful application of the opto-CD3ϵ-REACT approach to engineer opto-CD28-REACT suggests that this principle might also be applicable to other receptors—be it in immunology or other areas of biology or medicine. Expanding this approach to other stimulatory, co-stimulatory, or inhibitory receptors could provide powerful tools to deeply understand how cells interpret extracellular cues to control their activation, differentiation, or developmental processes.

## 4 Methods

### 4.1 Molecular cloning

The scFv targeting human CD28 was designed based on the sequence reported by Vanhove et al. (46). Synthetic DNA fragments codon-optimized for *E. coli* expression were ordered from Integrated DNA Technologies (IDT). The optimized scFv28.3 sequence was fused to monomeric oxidation-resistant green fluorescent protein (moxGFP) (31) and the first 100 amino acids of PIF6 (29). Constructs were assembled into expression vectors [pMH71 (24)/pRSET] using restriction enzyme digestion and ligation. All cloning steps were verified by DNA sequencing to ensure the accuracy of the final construct.

### 4.2 Protein production and purification

Opto-CD28-REACT and opto-CD3ϵ-REACT: The proteins were expressed in SHuffle T7 Express *E. coli* (NEB) overnight at 18 °C following induction with 0.2 mM IPTG. Cells were lysed by sonication, and the protein was purified through sequential chromatography steps, including Ni-affinity, anion exchange, and gel filtration chromatography. Ni-affinity purification was performed using a HisTrap FF 5-mL column equilibrated with Buffer A (50 mM Tris, 800 mM NaCl, 20 mM imidazole, and 10% glycerol, pH 8.0). Bound protein was eluted with 60% of Buffer B (50 mM Tris, 300 mM NaCl, 400 mM imidazole, and 10% glycerol, pH 8.0). Anion exchange chromatography was conducted using a MonoQ 5/50 GL column, with Buffer A (50 mM Tris, pH 8.5) for equilibration and Buffer B (50 mM Tris and 1 M NaCl, pH 8.0) for elution with a linear gradient. Final purification was achieved by gel filtration chromatography on a Superdex 200 10/300 column equilibrated with PBS.

Phytochrome B: The protein PhyB in biotinylated form was produced in *E. coli* from plasmid pMH1105 and purified by IMAC as described previously (47). This plasmid encodes the enzymes heme oxygenase 1 (HO1) and the PCB:ferredoxin oxidoreductase (PcyA), which are responsible for biosynthesis of the chromophore phycocyanobilin (PCB).

### 4.3 Protein characterization

SDS-PAGE and Coomassie stain: Protein purity was assessed by SDS-PAGE using a 10% polyacrylamide gel. Samples were boiled in Laemmli buffer at 95 °C for 5 min, separated alongside molecular weight markers, and stained with Coomassie Brilliant Blue R-250 to visualize protein bands.

Western blot: After SDS-PAGE, the separated proteins were transferred to a polyvinyl difluoride (PVDF) membrane by semi-dry blotting at 18 V for 50 min in semi-dry blotting buffer [0.29% (w/v) glycine, 0.048 M TrizmaBase, and 0.0375% SDS]. Membranes were blocked in wash buffer (PBS, 0.1% Tween 20) supplemented with 5% milk powder (w/v) and washed three times for 5 min. The primary antibodies anti-GFP (1:1,000, Rockland, Cat. No. 600-106-215) and anti-His (1:5,000, Thermo Fisher Scientific, Cat. No. MA1-21315-BTIN), diluted in wash buffer supplemented with 3% bovine serum albumin (BSA) and 0.02% NaN_3_, were incubated overnight at 4 °C. After washing, membranes were incubated for 45 min at room temperature, with either HRPO-conjugated anti-goat antibody (Thermo Fisher Scientific, Cat. No. 10466033) diluted 1:5,000 in blocking buffer or HRPO-conjugated anti-streptavidin antibody (Cell Signaling Technologies, Cat. No. 3999S) diluted 1:2,000 in wash buffer supplemented with 3% BSA and 0.02% NaN_3_. Proteins were detected via chemiluminescence with the ECL Prime Western Blotting Detection Reagent (Cytivia Amersham, Cat. No. 10308449).

### 4.4 Cell culture

Jurkat E6.1 cells were maintained in RPMI-1640 medium (Anprotec, Cat No. AC-LM-0058) supplemented with 10% (v/v) fetal bovine serum (FBS) (Sigma, Cat. No. F7524), 100 U/mL penicillin, and 100 µg/mL streptomycin as well as 10 mM HEPES (referred to as complete medium hereinafter) at 37 °C in a humidified atmosphere containing 5% CO_2_.

Primary human T cells were isolated from healthy human donor blood (ethics approval no. 22-1275-S1) via density gradient centrifugation using a ratio of 1:2.5 of Pancoll (Pan Biotech, Cat No. P04-60500) to donors’ blood (which was diluted 1:2 in PBS supplemented with 2 mM EDTA), followed by red blood cell lysis using ACK buffer (150 mM NH_4_Cl, 10 mM KHCO_3_, and 0.1 mM EDTA). Peripheral blood mononuclear cells (PBMCs) obtained from this stage were used for the stimulation and incubated at the same conditions and medium as used for Jurkat E6.1 cells.

### 4.5 Binding of opto-CD28-REACT to different cell types

A total of 1 × 10^5^ human T cells (Jurkat), murine T cells (2B4), or human B cells (Nalm6) were incubated with 100 mM opto-CD28-REACT for 30 min at 37 °C in a humidified atmosphere containing 5% CO_2_. As a control, cells were incubated with 1:200 diluted biotin-conjugated CD28 antibody (BioLegend, Cat. No. 102103) for 15 min on ice followed by a secondary staining with APC-conjugated streptavidin (BioLegend, Cat. No. 405243). After washing, cells were resuspended in staining buffer (PBS + 1% FBS) and analyzed using an Attune NxT flow cytometer. GFP and APC-conjugates were excited with a 488- and 637-nm laser and detected using a 530/30- and 670/14-nm emission filter, respectively.

### 4.6 Binding competition between opto-CD28-REACT and an anti-CD28 antibody

A total of 1 × 10^5^ Jurkat T cells per condition were incubated with 100 nM opto-CD28-REACT and different concentrations of either anti-CD28 antibody (BioLegend, Cat. No. 302902) or a non-competitive antibody (anti-HA-tag, BioLegend, Cat. No. 901505) for 30 min on ice. After incubation, cells were resuspended in staining buffer (PBS + 1% FBS) and analyzed using an Attune NxT flow cytometer. GFP was excited with a 488-nm laser and detected using a 530/30-nm emission filter.

### 4.7 PhyB-coupled beads

All steps involving PhyB were performed under green light conditions to prevent premature activation. The streptavidin-coated bead (Spherotec, Cat. No. SVP5-60-5) suspension was thoroughly vortexed to ensure uniformity. A portion of the bead solution was transferred to a reaction tube and diluted 1:1 with PBS containing 1.5 mM TCEP. The mixture was centrifuged at 15,000 × *g* for 10 min at 4 °C, after which the supernatant was carefully discarded. This washing step was repeated. For PhyB coating, beads were incubated with 60 µg/mL of PhyB in PBS containing 1.5 mM TCEP at a concentration of 1 × 10^6^ beads in 50 µL on ice in the dark for 1 h. After washing, beads were resuspended in biotin-free RPMI-1640 (Biomol, Cat. No. R9002-01) complete medium at a concentration of 1 × 10^5^ beads in 50 or 100 µL. For initial stimulation, 1 × 10^5^ beads were added to each designated well, and the plate was illuminated with the 740-nm light for 1 min to inactivate the PhyB-coated beads. A detailed protocol can be found in (48).

### 4.8 Light-dependent activation of Jurkat T cells

Jurkat T cells were passaged 1:2 the day before the experiment. To prepare cells for optogenetic stimulation, the indicated amount of opto-CD3ϵ-REACT and/or opto-CD28-REACT was given to 1 × 10^6^ cells/mL in biotin-free RPMI-1640 (Biomol, Cat. No. R9002-01) complete medium. For the antibody stimulation, 1 µg/mL of soluble anti-CD3 (UCHT1) and/or anti-CD28 (BioLegend, Cat. No. 302902) was added to the cells, respectively. The final volume of each well was 225 µL in a black 96-well plate with a transparent bottom (Greiner, Cat. No. 655090). Stimulation was conducted for 24 h at 37 °C in a humidified atmosphere containing 5% CO_2_. Afterwards, supernatants were frozen at −20 °C and cells were stained with AF647-conjugated CD69 antibody (Thermo Fisher Scientific, Cat. No. MHCD6905) and PE-conjugated anti-CD25 antibody (Thermo Fisher Scientific, Cat. No. MHCD2504) diluted 1:200 in staining buffer (PBS, 1% FBS) for 15 min on ice. After washing, cells were resuspended in staining buffer and analyzed using an Attune NxT flow cytometer. GFP, PE, and AF647 conjugates were excited with a 488-, 561-, and 637-nm laser and detected using a 530/30-, 585/16-, and 670/14-nm emission filter, respectively. Data were analyzed using the gating strategy displayed in **Supplementary Figure S2**.

### 4.9 Intracellular phospho-ERK staining after optogenetic activation of Jurkat T cells

5 × 10^4^ opto-APCs (27, 49) in 100 µl RPMI-1640 medium supplemented with 1% (v/v) FBS, 100 U/ml penicillin, and 100 μg/ml streptomycin and 10 mM HEPES were seeded in one well of a black 96-well plate with a transparent bottom (Greiner, Cat. No. 655090) and incubated overnight at 37 °C in a humidified atmosphere containing 5% CO_2_. Opto-CD28-REACT (30 nM) was given to 1 × 10^6^ Jurkat cells/mL in RPMI-1640 medium without FBS and incubated for 1 h at 37 °C in a humidified atmosphere containing 5% CO_2_. In the meantime, the medium from the opto-APCs was removed and 50 µL of 10 µg/mL of PhyB-mCherry-SpyTag containing RPMI-1640 medium without FBS was added per well and incubated for 30 min at 37 °C in a humidified atmosphere containing 5% CO_2_. The excess PhyB-mCherry-SpyTag solution was removed before adding 50 µL of the T cells with the opto-CD28-REACT. In addition, a final concentration of 50 ng/mL of soluble anti-CD3 (UCHT1) was added to the cells. The final volume of each well was 100 µL. Stimulation was conducted for up to 16 min at 37 °C in a humidified atmosphere containing 5% CO_2_. To stop the stimulation, cells were transferred into a 96V-bottom plate containing 150 µL of 4% paraformaldehyde (Sigma-Aldrich, Cat. No. 30525-89-4) and incubated on ice for 30 min. Afterwards, cells were pelleted for 5 min, at 4 °C and 550 × *g*, and washed with staining buffer (PBS, 1% FBS). For permeabilization, cells were resuspended in 50 µL of ice-cold 90% methanol (Carl Roth, Cat. No. 4627.6) and incubated for 30 min on ice. After washing, cells were stained with the anti-phospho-ERK antibody (Cell Signaling Technology, Cat. No. 9101) diluted 1:200 in staining buffer and incubated overnight at 4 °C. After washing, cells were stained with a secondary goat-anti-rabbit DyLight-633 antibody (Invitrogen, Cat. No. 35563) diluted 1:200 in staining buffer for 2 h at room temperature. After washing, cells were resuspended in staining buffer and analyzed using an Attune NxT flow cytometer. BFP, mCherry, and DyLight-633 conjugates were excited with a 405-, 561-, and 637-nm laser and detected using a 512/15-, 620/15-, and 670/14-nm emission filter, respectively. Data were analyzed using the gating strategy displayed in **Supplementary Figure S3**.

### 4.10 Light-dependent activation of primary human T cells

A total of 1 × 10^6^ PBMCs/mL in biotin-free RPMI-1640 medium (Biomol, Cat. No. R9002-01) supplemented with 100 U/mL penicillin, 100 µg/mL streptomycin, and 10 mM HEPES were incubated with 1:1,000 dilution of CTV (Invitrogen, Cat. No. C34557) for 20 min at 37 °C in a humidified atmosphere containing 5% CO_2._ To remove any free dye, biotin-free FBS was added to a final concentration of 10% and further incubated for 5 min. After washing, 5 mM of opto-CD3ϵ-REACT and/or 30 mM opto-CD28-REACT was given to a concentration of 1 × 10^6^ PBMCs/mL in biotin-free RPMI-1640 complete medium. Cells were incubated at 37 °C in a humidified atmosphere containing 5% CO_2_ for 30 min. A total of 1.5 × 10^5^ cells were seeded per well of a black 96-well plate with a transparent bottom (Greiner, Cat. No. 655090). For the antibody stimulations, wells were previously coated with 50 µL of PBS containing 1 µg/mL of anti-CD3 (UCHT1) and anti-CD28 (BioLegend, Cat. No. 302902), and incubated at 37 °C in a humidified atmosphere containing 5% CO_2_ for 2 h. PBMCs used for controls were not coupled to opto-REACTs. The final volume at the start of each stimulation was 200 μL. After 24 and 48 h, 50 μL of biotin-free RPMI complete medium was added to every well. Optogenetic stimulation was conducted up to 72 h at 37 °C in a humidified atmosphere containing 5% CO_2_. Afterwards, supernatants were frozen at −80 °C and cells were stained with Zombie NIR (BioLegend, Cat. No. 423105) diluted 1:500 in PBS at room temperature for 20 min in the dark. After washing, antibody staining was performed with AF488-conjugated CD3 antibody (BioLegend, Cat. No. 300415), AF700-conjugated CD8 antibody (Beckman Coulter, Cat. No. B76279), AF647-conjugated CD69 antibody (Thermo Fisher Scientific, Cat. No. MHCD6905), and PE-conjugated anti-CD25 antibody (Thermo Fisher Scientific, Cat. No. MHCD2504) diluted 1:200 in staining buffer (PBS, 1% FBS) for 15 min on ice. After washing, cells were resuspended in staining buffer and analyzed using an Attune NxT flow cytometer. CTV; GFP; AF488, PE, AF647, and AF700 conjugates; and NIR were excited with a 405-, 488-, 561-, and 637-nm laser and detected using 440/50-, 530/30-, 585/16-, 670/14-, 720/30-, and 780/60-nm emission filters, respectively. Data were analyzed using the gating strategy displayed in **Supplementary Figure S4A**.

### 4.11 Illumination

Cells were illuminated using optoPlate-96 (50). Jurkat T cells were illuminated with a 630-nm light [30 s, 540 µEinstein (µE ≙ µmol m^−2^ s^−1^) followed by 14.5 min dark] and a 780-nm light (1 min, 1,200 µE followed by 14 min dark). Primary human T cells were illuminated with 630 nm (30 s, 270 µE followed by 14.5 min dark) or pulsed with 780 nm (1 min, 600 µE followed by 14 min dark). Light-emitting diodes (LEDs) were programmed using optoConfig-96 (51).

### 4.12 ELISA

IL-2 levels in Jurkat T-cell supernatants were quantified using an IL-2 ELISA kit according to the manufacturer’s protocol (Thermo Fisher Scientific, Cat. No. 88-7025-88). To increase sensitivity, all incubation steps were performed overnight. Absorbance at 450 and 570 nm was measured using a MultiskanGo microplate spectrophotometer (Thermo Fisher Scientific), and background subtraction (450–570 nm) followed by subtraction of the blank wells was applied.

IL-2 and IFN-γ levels in primary human T-cell supernatants were quantified using an IL-2 (BioLegend, Cat. No. 431804) or IFN-γ (BioLegend, Cat. No. 430104) ELISA kit according to the manufacturer’s protocol. Absorbance at 450 nm was measured and blank subtraction was applied.

### 4.13 Quantification and statistical analysis

Figures and figure legends provide statistical parameters such as the actual value of *n*, precision measurements, and statistical significance. Datasets were tested for normality using the Shapiro–Wilk test. For experiments displayed in **Figures 1**–**4**, a one-way analysis of variance (ANOVA) with Dunnett’s multiple comparisons was performed. For experiments shown in **Figure 5**, **Supplementary Figure S6**, and **Figure 6**, a two-way ANOVA with Turkey’s multiple comparisons was performed. For experiments shown in **Figure 7** and **Supplementary Figure S7**, a one-way ANOVA with Friedman’s multiple comparisons was performed. Statistical analysis was performed in GraphPad PRISM 9.5.1. Significance thresholds: ns: *p* > 0.05; *p < 0.05; **p < 0.01; ***p < 0.001; ****p < 0.0001.

### 4.14 Software

Flow cytometry data were analyzed with FlowJo (v10.10.0, Becton, Dickinson and Company), and statistical analysis and plotting were performed in GraphPad Prism (v9.5.1, GraphPad Software). Western blot images were edited with Adobe Photoshop 2023. **Figures 1A, B**, **2A**, and **4A** were created in BioRender.com.

## Data Availability

The original contributions presented in the study are included in the article/**Supplementary Material**. Further inquiries can be directed to the corresponding author.
