# Supplementary material for: Opto-CD28-REACT: optogenetic co-stimulatory receptor activation on non-engineered human T cells
Source: Front Immunol. 2025 Sep 19;16:1646135. doi: 10.3389/fimmu.2025.1646135 (PMC12491302; doi:10.3389/fimmu.2025.1646135)
Supplement: Supplementary file 1 [file DataSheet1.pdf]

Supplementary Material

A

Nucleic acid sequence of opto-CD28-REACT:

T7 promoter

CD28 scFv

moxGFP

PIF6(1-100, C9S, C10S)

His6

TAATACGACTCACTATAGGGAGACCACAACGGTTTCCCTCTAGAAATAATTTTGTTTAACTTTAAGAAGGAGATATACATAT
GGTCAAACTTCAGCAAAGCGGAGCAGAATTGGTCAAACCCGGTGCCTCCGTACGTCTGTCTTGTAAAGCAAGCGGCTAC
ACGTTCACGGAATACATTATTCATTGGATCAAGCTGCGTAGCGGGCAAGGGCTGGAGTGGATCGGGTGGTTTTATCCAG
GGAGTAATGACATCCAATATAATGCAAAATTTAAGGGAAAAGCAACTCTTACGGCTGACAAGAGCTCCAGCACGGTTTAT
ATGGAATTAACAGGTCTTACGAGTGAGGATTCCGCCGTGTATTTTTGCGCTCGTCGTGATGACTTCTCCGGGTATGATGC
ATTGCCCTACTGGGGCCAGGGAACGATGGTGACGGTAAGCTCAGGTGGTGGTGGCTCTGGTGGCGGTGGTTCAGGCG
GTGGCGGTAGCGACATCCAAATGACTCAAAGCCCCGCTTCGCTGTCTGTTTCTGTTGGGGAGACAGTAACCATTACCTG
CCGTACTAATGAGAACATCTATTCTAATCTGGCCTGGTATCAGCAAAAACAGGGGAAATCTCCTCAATTACTGATTTACGC
TGCGACCCACCTTGTCGAAGGTGTGCCCAGCCGTTTTAGCGGTAGTGGTAGTGGGACCCAGTACAGCTTAAAGATTACC
TCACTTCAGTCGGAAGACTTTGGGAACTACTATTGCCAACATTTCTGGGGAACGCCATGTACCTTTGGGGGTGGGACGA
AGTTGGAGATCAAGCGGGGTAGTGCAGGTTCTGCGGGTGGCGTGTCCAAGGGCGAGGAGCTGTTCACCGGGGTGGTG
CCCATCCTGGTCGAGCTGGACGGCGACGTAAACGGCCACAAGTTCTCCGTGCGGGGCGAGGGCGAGGGCGATGCCA
CCAACGGCAAGCTGACCCTGAAGTTCATCAGCACCACCGGCAAGCTGCCCGTGCCCTGGCCCACCCTCGTGACCACCC
TGACCTACGGCGTGCAGAGCTTCTCCCGCTACCCCGACCACATGAAGCGCCACGACTTCTTCAAGAGCGCCATGCCCG
AAGGCTACGTCCAGGAGCGCACCATCTCCTTCAAGGACGACGGCACCTACAAGACCCGCGCCGAGGTGAAGTTCGAGG
GCGACACCCTGGTGAACCGCATCGAGCTGAAGGGCATCGACTTCAAGGAGGACGGCAACATCCTGGGGCACAAGCTG
GAGTACAACTTCAACTCCCACAACGTCTATATCACCGCCGACAAGCAGAAGAACGGCATCAAGGCCAACTTCAAGATCC
GCCACAACGTGGAGGACGGCTCCGTGCAGCTCGCCGACCACTACCAGCAGAACACCCCCATCGGCGACGGCCCCGTG
CTGCTGCCCGACAACCACTACCTGTCCACCCAGTCCAAGCTGTCCAAAGACCCCAACGAGAAGCGCGATCACATGGTCC
TTCTGGAGTTCGTGACCGCCGCCGGGATCACTCACGGCATGGACGAGCTGTACAAGGGCTCCGCAGGTTCTGCTGGTA
TGATGTTCTTACCAACCGATTACTCGAGCAGGTTAAGCGATCAAGAGTATATGGAGCTTGTGTTTGAGAATGGCCAGATT
CTTGCAAAGGGCCAAAGATCCAACGTTTCTCTGCATAATCAACGTACCAAATCGATCATGGATTTGTATGAGGCAGAGTA
TAACGAGGATTTCATGAAGAGTATCATCCATGGTGGTGGTGGTGCCATCACAAATCTCGGGGACACGCAGGTTGTTCCA
CAAAGTCATGTTGCTGCTGCCCATGAAACAAACATGTTGGAAAGCAATAAACATGTTGACCATCATCACCATCACCATTAA

B

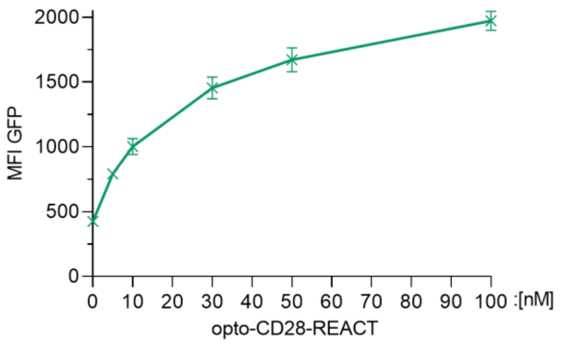

Supplementary Figure 1: DNA sequence and CD28-binding of opto-CD28-REACT

(A) Nucleic acid sequence of opto-CD28-REACT plasmid generated in this study. T7 promotor highlighted in grey, heavy and light chain of CD28 shown in turquoise, moxGFP displayed in green, PIF6 shown in yellow and His6-tag in purple. **(B)** Titration of opto-CD28-REACT binding to CD28-positive Jurkat T cells in a flow cytometry experiment as in Figure 1E. n = 2, each in technical triplicates. Error bars represent SEM. MFI, median fluorescence intensity.

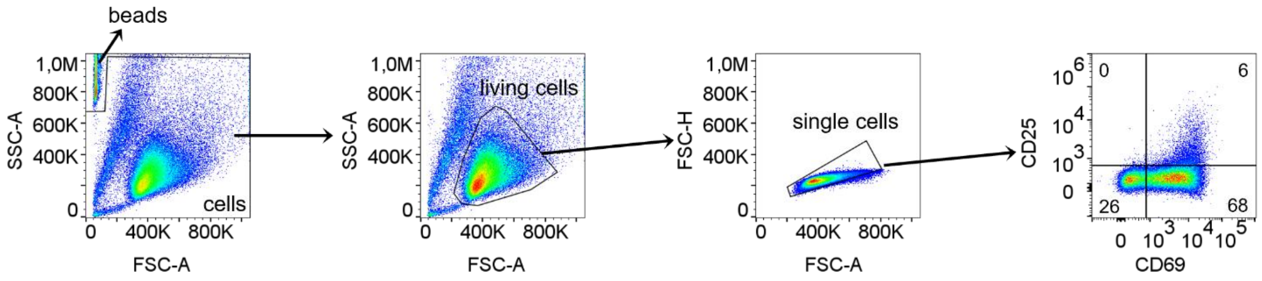

Supplementary Figure 2: Gating of the Jurkat cells in the co-culture with the PhyB beads after the flow cytometry measurement

The gating strategy of a representative sample from experiments performed with Jurkat T cells stimulated with anti-CD3 antibodies and opto-CD28-REACT via PhyB-bound beads. First, the beads were excluded from the further analysis. Then living Jurkat cells were gated based on the FSC-A and SSC-A signal before excluding doublets based on the FSC-H and FSC-A signal. Then, PE- (CD25) and AF647- (CD69) positive cells were identified based on the negative control. Data correspond to Figure 2. FCS-A, forward scatter area; FCS-H, forward scatter hight; SSC-A, side scatter area.

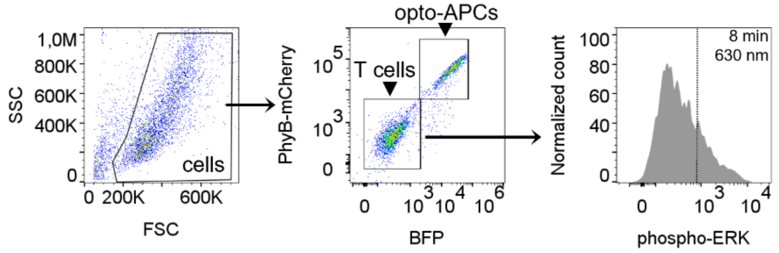

Supplementary Figure 3: Gating of the Jurkat cells in the co-culture with opto-APCs after the flow cytometry measurement

The gating strategy of a representative sample from experiments performed with Jurkat T cells stimulated with anti-CD3 antibodies and opto-CD28-REACT via opto-APCs. First, cells were gated based on the FSC and SSC signal. The opto-APCs express the blue fluorescent protein (BFP) and the loaded PhyB is coupled to mCherry. Hence, T cells were gated based on being double-negative for BFP and mCherry. Finally, the fluorescence intensity of the anti-phospho-ERK antibody stain (DyLight633) was examined and phospho-ERK positive cells were identified based on the fluorescence of the unstimulated cells. Dashed line indicates separation of positive and negative cells. Data correspond to Figure 3E. FCS, forward scatter; SSC, side scatter.

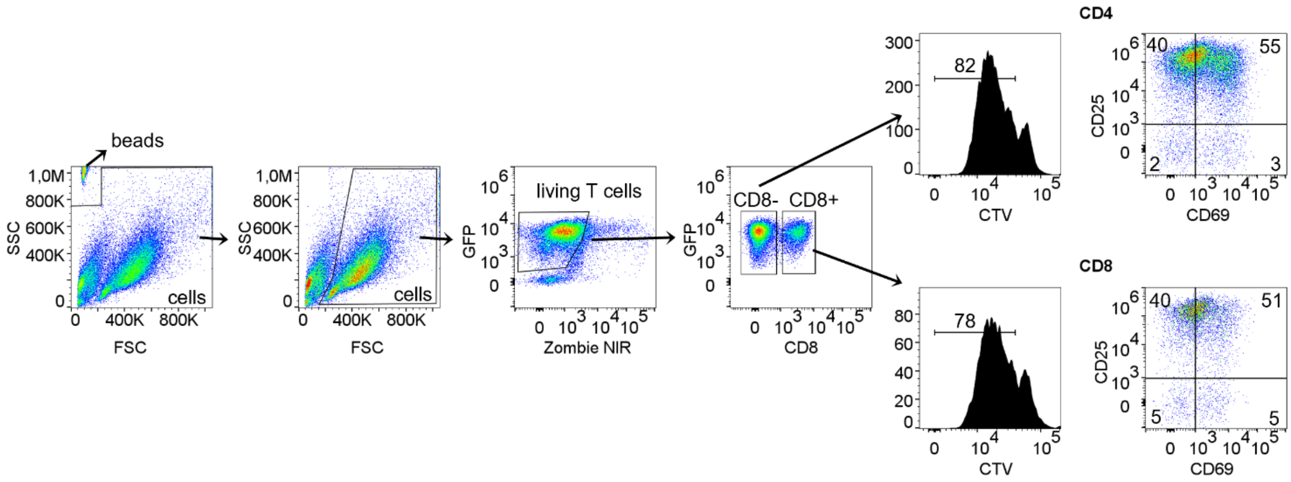

Supplementary Figure 4: Gating of primary human T cells in the co-culture with the PhyB beads after the flow cytometry measurement

The gating strategy of a representative sample from experiments performed with primary human T cells stimulated with 5 nM opto-CD3-REACT and 30 nM opto-CD28-REACT via PhyB-bound beads and stained with the dead cell dye Zombie NIR, anti-CD3-AF488, anti-CD8-AF700, anti-CD25-PE and anti-CD69-AF647 antibodies is depicted. First, the beads were excluded from further analysis. Then cells were gated based on FSC and SSC signal before selecting living T cells based on GFP/AF488 and zombie NIR signal. $CD8^+$ and $CD8^-$ T cells (the latter representing the CD4 cells) were separated based on anti-CD8-AF700 staining. Afterwards cells were analyzed separately for CTV-negative (proliferation), PE-positive (CD25) and AF647-positive (CD69) cells were identified based on the negative control. Data correspond to Figure 5, S5 and S6.

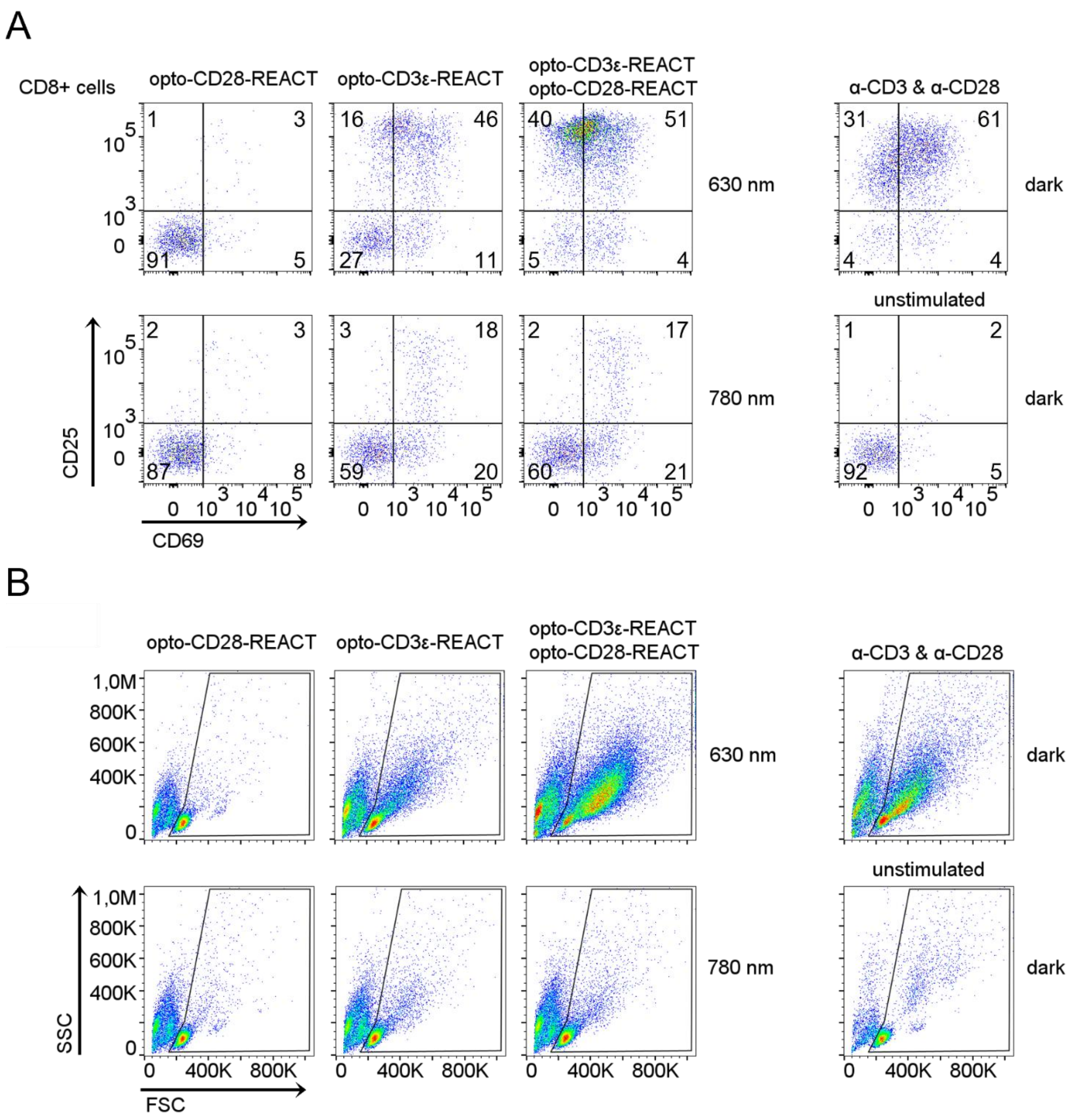

Supplementary Figure 5: Optogenetic activation of primary human CD8^{+} T cells via CD28 and TCR

Selected flow cytometry dot plots of the experiment shown in Figure 5.

(A) Dot plots of the living CD8^{+} cells of one donor is shown, displaying CD25-PE and CD69-AF647 fluorescence intensities. Stimulations are indicated on the top of each panel, and light conditions to the right. **(B)** As in (A) showing the forward scatter (FSC) and side scatter (SSC) of all cells.

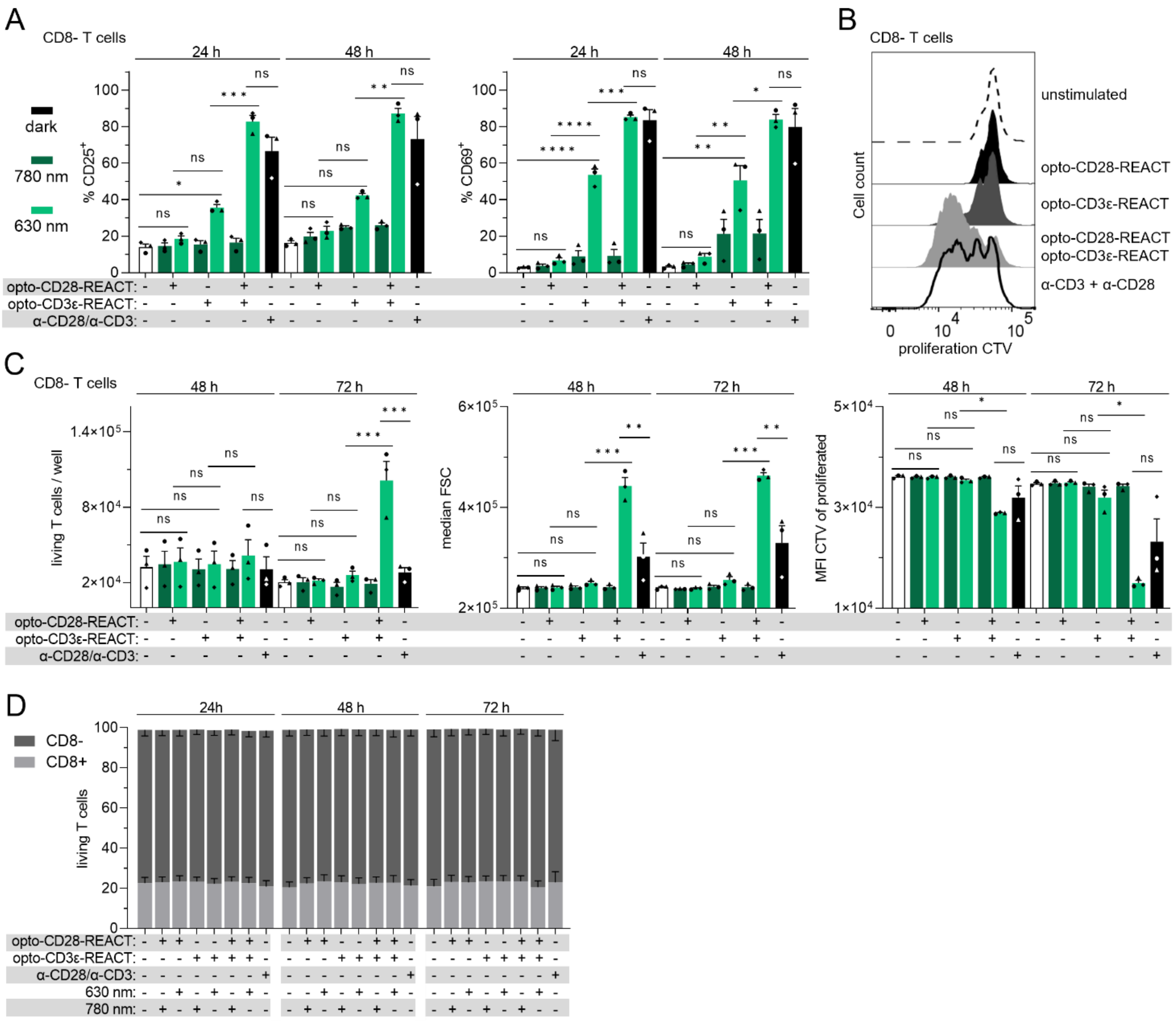

Supplementary Figure 6: Optogenetic activation of primary human CD8$^-$ T cells via CD28 and TCR

In contrast to Figure 5, the samples shown here display CD8$^-$ cells. After optogenetic stimulation and flow cytometry, cells were gated on the CD8$^-$ (i.e., CD4$^+$) T cell population, rather than on the CD8$^+$ cells as in Figure 5. Cells were treated as described in Figure 5. **(A)** Bar diagrams show the percentage of CD25$^+$ (left panel) and CD69$^+$ (right panel) cells among CD8$^-$ living T cells after 24 and 48h. **(B)** Flow cytometry histograms show the proliferation of CD8$^-$ living T cells stained with CellTrace Violet (CTV) after 72h. **(C)** Bar diagrams show the number of living CD8$^-$ T cells per well, the size of CD8$^-$ T cells (median FSC), and the MFI of the proliferated CD8$^-$ cells after 48 and 72 h. **(D)** Bar diagrams show the percentages of CD8$^+$ and CD8$^-$ of the conditions form experiments described in Figure 5 and S6. All experiments depicted in this figure are n = 3 healthy donors, each in technical duplicates. All 630 nm treated, unstimulated and antibody treated samples of every time point ware compared with every other for statistical analysis. Error bars represent SEM. FSC, forward scatter; CTV, CellTrace Violet; MFI, median fluorescence intensity.

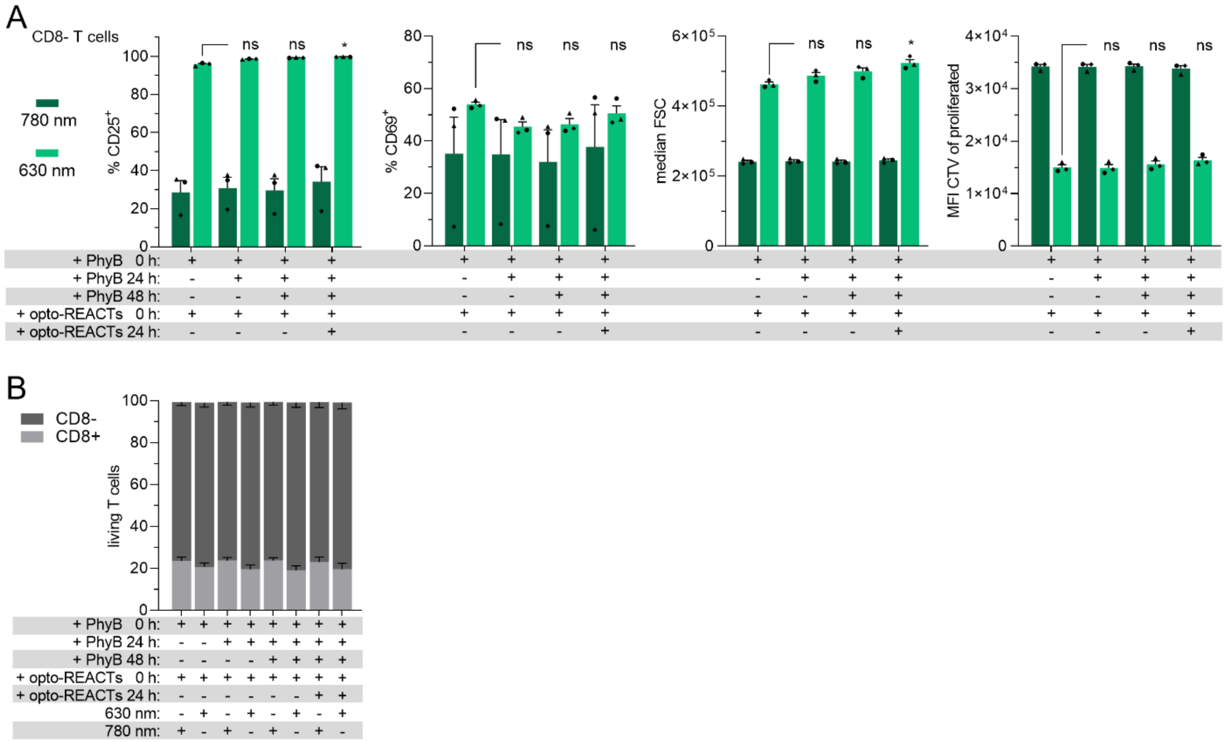

Supplementary Figure 7: Adding of additional opto-REACTs and PhyB does not enhance activation of $CD8^-$ primary T cells

(A) Cells were treated as described in Figure 7. The gating strategy in given in Figure S3. Bar diagrams of $CD8^-$ T cells show from left to right, percentage of $CD25^+$, $CD69^+$, size of cells (median FSC) and MFI of proliferated cells. **(B)** Bar diagrams show the percentages of $CD8^+$ and $CD8^-$ of the conditions form experiments described in Figure 7 and S7. All experiments depicted in this figure are n=3 healthy donors, each in technical duplicates. Only samples treated with 630 nm light were taken into account for statistical analysis. Furthermore, samples in which PhyB and/or opto-REACTs were added after certain time periods were compared, for statistical analysis, to conditions where PhyB and opto-REACTs were only added at the beginning of the experiment. Error bars represent SEM. FCS, forward scatter; CTV, CellTrace Violet; MFI, median fluorescence intensity.
